# Functional importance of NUDT9H domain and N-terminal ADPR-binding pocket in two species variants of vertebrate TRPM2 channels

**DOI:** 10.1038/s41598-019-55232-5

**Published:** 2019-12-16

**Authors:** Frank J. P. Kühn, Wiebke Ehrlich, Daniel Barth, Cornelia Kühn, Andreas Lückhoff

**Affiliations:** Institute of Physiology, Medical Faculty, RWTH Aachen, D52057 Aachen, Germany

**Keywords:** Biochemistry, Cell biology, Molecular biology, Physiology, Molecular biophysics, Biophysics, Membrane biophysics, Ion transport

## Abstract

There are at least two different principles of how ADP-ribose (ADPR) induces activation of TRPM2 channels. In human TRPM2, gating requires the C-terminal NUDT9H domain as ADPR-binding module, whereas in sea anemone, NUDT9H is dispensable and binding of ADPR occurs N-terminally. Zebrafish TRPM2 needs both, the N-terminal ADPR-binding pocket and NUDT9H. Our aim was to pinpoint the relative functional contributions of NUDT9H and the N-terminal ADPR-binding pocket in zebrafish TRPM2, to identify fundamental mechanisms of ADPR-directed gating. We show that the NUDT9H domains of human and zebrafish TRPM2 are interchangeable since chimeras generate ADPR-sensitive channels. A point mutation at a highly conserved position within NUDT9H induces loss-of-function in both vertebrate channels. The substrate specificity of zebrafish TRPM2 corresponds to that of sea anemone TRPM2, indicating gating by the proposed N-terminal ADPR-binding pocket. However, a point mutation in this region abolishes ADPR activation also in human TRPM2. These findings provide functional evidence for an uniform N-terminal ADPR-binding pocket in TRPM2 of zebrafish and sea anemone with modified function in human TRPM2. The structural importance of NUDT9H in vertebrate TRPM2 can be associated with a single amino acid residue which is not directly involved in the binding of ADPR.

## Introduction

The Melastatin Type 2 cation channel (TRPM2) represents a unique member of the family of Transient Receptor Potential (TRP) cation channels. This is reflected in its diverse physiological functions (e.g. reviewed by refs. ^1–4^), its exceptional gating mechanism^5,6^ as well as its unusual species-specific variations^7–10^. TRPM2 forms a chimera of ion channel and enzyme domain, the latter being homologous to the human ADP-ribose pyrophosphatase NUDT9^5,11^.

The nicotinamide adenine dinucleotide (β-NAD^+^) metabolite ADP-ribose (ADPR) has become the subject of intensive research efforts in connection with the post-translational modification of proteins. The enzymatic addition of an ADPR molecule to a target substrate referred to as ADP-ribosylation represents one of several processes involved in the epigenetic regulation of the proteome (e.g. reviewed by ref. ^12^). The ADPR-dependent activation of TRPM2, however, does not take place via ADP-ribosylation but rather through a different type of interaction in which the C-terminal NUDT9H domain represents a key player, at least in some species variants including the human orthologue^5,6,8,13–16^.

It is generally accepted that in human TRPM2 (hTRPM2), ADPR binds to the NUDT9H domain of the channel^17,18^, thereby inducing conformational changes that results in the opening of the pore^6^. During this interaction there is no enzymatic cleavage of ADPR, because the NUDT9H domain of hTRPM2 lacks significant catalytic activity^15,16^ and channel activation is also induced with the non-cleavable ADPR-analogue Alpha-Beta Methylene ADPR (AMPCPR)^16^.

Surprisingly, recent findings indicate that the ADPR-dependent gating mechanism of TRPM2 has been significantly modified during evolution. The functional analysis of a far-distantly related species variant from the sea anemone *Nematostella vectensis* (nvTRPM2) revealed that ADPR-dependent channel gating is also possible in the complete absence of the endogenous NUDT9H domain^15,19^. These findings directly imply the presence of an additional ADPR interaction site located in the ion channel domain of nvTRPM2^7,15^. In further experiments it was demonstrated that both ADPR interaction sites of nvTRPM2 show strikingly different substrate specificities^9^ and that the NUDT9H domain of nvTRPM2 has robust ADPRase activity^9,10,15^ which possibly is correlated with a regulatory function *in vivo*^7^. Thus, for the TRPM2 orthologues of sea anemone and human, the experimental data strongly suggest the existence of two principally different ADPR-dependent gating mechanisms.

The recent structural and functional characterization of a further TRPM2 orthologue from the zebrafish *Danio rerio* (drTRPM2) proposed an N-terminal ADPR-binding pocket crucial for ADPR-directed channel gating^8^. However, in this species variant, the presence of the NUDT9H domain is still indispensable for channel function, although its binding affinity to ADPR is greatly reduced^6,8^.

The question now arises which further property of NUDT9H determines its essential role in the gating process of drTRPM2. Obviously, there are key differences between hTRPM2 and drTRPM2 with regard to the functional interactions between the NUDT9H domain and the rest of the channel^6,20^. Most importantly, the putative N-terminal ADPR-binding pocket of drTRPM2 is almost completely conserved in nvTRPM2 as well as in hTRPM2. This finding raises further questions because for hTRPM2, there is experimental evidence that this region is not important for channel gating^6^. The aim of the current study was to characterize the ADPR-dependent gating mechanisms of drTRPM2 in more detail in order to allow a clear assignment to one of the two principal categories, i.e. nvTRPM2 or hTRPM2. Furthermore, we wished to explore to what extent the different gating mechanisms are compatible with each other in order to pinpoint fundamental gating parameters of ADPR-directed gating in TRPM2 channels.

The current study reveals that the substrate specificity of drTRPM2 matches with nvTRPM2 with respect to the ADPR-analogues IDP-ribose (IDPR), 8-(thiophen-3-yl)-ADPR (8-TP-ADPR) and 8-(3-acetylphenyl)-ADPR (8-(3AP)-ADPR) suggesting an uniform N-terminal ADPR interaction site. In contrast to hTRPM2 which is inhibited by 2-Aminoethoxy diphenyl borate (2-APB), the drTRPM2 channel is shown to be activated by 2-APB in a similar manner as previously demonstrated for nvTRPM2^21^. Both in drTRPM2 and in nvTRPM2 the mutation of the putative N-terminal ADPR interaction site suppressed not only the sensitivity to ADPR but also that to 2-APB. Moreover, the novel ADPR interaction site is crucial for hTRPM2 as well. On the other hand, chimeras of hTRPM2 and drTRPM2 where the NUDT9H domains have been swapped show ADPR-dependent gating; moreover, the same single point mutation within a conserved region of NUDT9H prevents ADPR-dependent gating both in hTRPM2 and in drTRPM2. Our study provides important new insights about critical factors of ADPR-dependent channel gating.

## Results

### Basic characteristics of drTRPM2 currents stimulated by ADPR and H_2_O_2_

**Figure 1:**
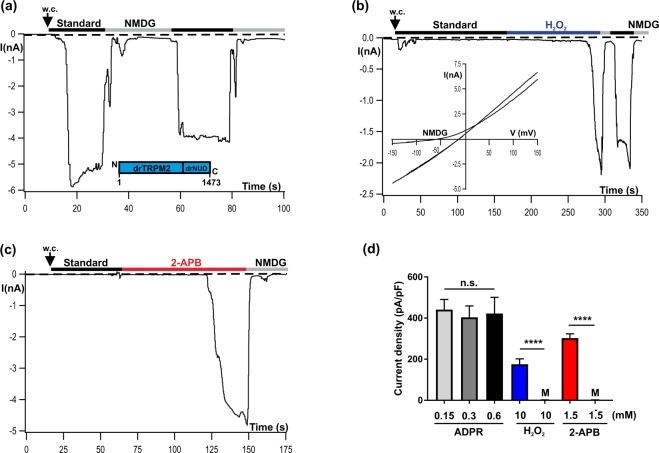
Activation of drTRPM2 by ADPR, H_2_O_2_ and 2-APB. Representative whole-cell patch-clamp experiments of HEK-293 cells heterologeously expressing drTRPM2 (**a**) ADPR (0.15 mM) was infused into the cell through the patch pipette together with 1 µM Ca^2+^. Current onset occurs with a short delay after reaching whole-cell configuration (w.c.). Substitution of external Na^+^ in the standard bath solution (indicated with black bars, ref. to Methods) with the impermeable cation NMDG (indicated with gray bars) blocks the inward currents. (**b**) Same pipette solution as used in panel a but without ADPR. Activation is induced after extracellular application of 10 mM H_2_O_2_ to the standard bath solution (indicated with blue bar). Note the delayed time course of activation. The corresponding current-voltage relation, as obtained with voltage-ramps, is given in the inset. (**c**) Same pipette solution as used in panel b. Stimulation was performed by superfusion of the cells with standard bath solution containing 1.5 mM 2-APB (indicated with red bar). There was no noticeable current decline unless the cells were superfused with NMDG bath solution. (**d**) Summary of the effects of different agonists on drTRPM2 including control experiments (M, Mock-transfected cells) either performed with ADPR or with 2-APB. All data are presented as mean ± s.e.m. Differences are significant at ****(P < 0.0001) evaluated with an unpaired Student’s t-test, n = 4–16. n.s., not significant.

During heterologous expression in HEK-293 cells the whole-cell currents of the TRPM2 orthologues of human (hTRPM2) and sea anemone (nvTRPM2) display distinct characteristics in the presence of ADPR (pipette solution) or H_2_O_2_ (bath solution)^19^. We tested the TRPM2 orthologue of zebrafish (drTRPM2) under identical experimental conditions i.e. with a moderately high (1 µM) concentration of Ca^2+^ in the patch-pipette. When ADPR (0.15 mM) was intracellularly applied through the patch pipette, a current developed gradually and reached a maximum within about half a minute. There was little inactivation of the current that remained nearly constant over several minutes (Fig. 1a). Extracellular hydrogen peroxide (H_2_O_2_, 10 mM) induced currents consistently, although only after a characteristic delay of several minutes **(**Fig. 1b**)**. The current-voltage relation was almost linear and the inward component could be blocked by substitution of extracellular cations with the impermeable cation NMDG (inset of Fig. 1b). The results are closely similar to those previously obtained on hTRPM2 and distinctly different from those of nvTRPM2^19^.

### 2-APB induces currents through drTRPM2

By using the non-specific TRP channel modulator 2-APB, hTRPM2 and nvTRPM2 can be clearly distinguished^21^. Whereas nvTRPM2 is strongly activated during stimulation with higher concentrations (extracellular ≥1 mM) of 2-APB, the human orthologue is completely inhibited already at submillimolar concentrations of 2-APB^21,22^. When HEK-293 cells expressing wild-type drTRPM2 were stimulated with 2-APB (1.5 mM added to the bath) large currents developed (Fig. 1c) showing the same characteristics as described for nvTRPM2^21^. In particular there was no noticeable current inactivation in the presence of 2-APB. The effects of the three stimuli ADPR, H_2_O_2_, and 2-APB on wild-type drTRPM2 are summarized in Fig. 1d, along with those on controls (mock-transfected HEK-293 cells). As can be also derived from the bar chart, currents of drTRPM2 reached maximum amplitudes already in the presence of 0.15 mM ADPR which could not be significantly enhanced with higher concentrations of ADPR.

### Inside-out patch clamp recordings reveal distinct characteristics of TRPM2 orthologues

**Figure 2:**
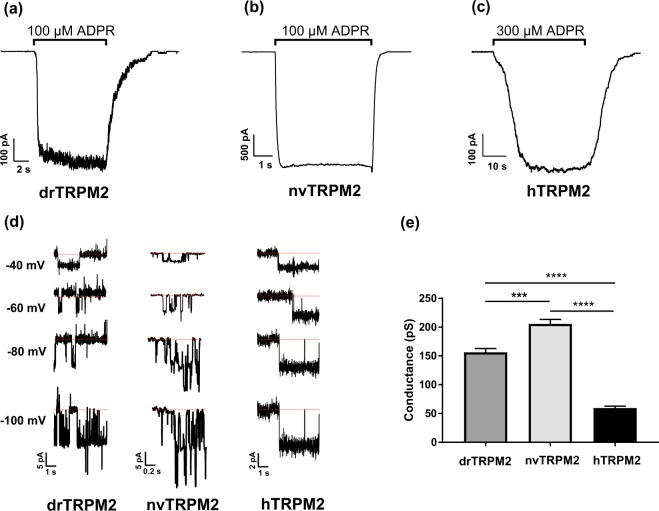
Typical kinetics of ADPR-dependent currents of drTRPM2, nvTRPM2 and hTRPM2 as obtained from inside-out patches. (**a**–**c**) Representative current traces of HEK-293 cells expressing the respective TRPM2 orthologue as indicated. Stimulation was performed with a bath solution containing Ca^2+^ (1 µM) and ADPR (as indicated). In the presence of ADPR the currents of drTRPM2 and nvTRPM2 developed almost instantaneously and quickly returned to baseline after ADPR removal. In contrast, in hTRPM2 the time course of both on- and off-kinetics is markedly delayed during application and withdrawal of ADPR (note the different time scales). Values are given in Table 1. (**d**) Single channel current amplitudes at indicated voltages were used to determine the conductance of drTRPM2, nvTRPM2 and hTRPM2 (**e**). Conductance significantly and gradually increases from hTRPM2 to drTRPM2 and nvTRPM2. All data are presented as mean ± s.d. differences are significant at ***(P < 0.001) and ****(P < 0.0001) evaluated with a one-way ANOVA and the Bonferroni correction, n = 3.

The characteristics of drTRPM2 single channels in comparison to the sea anemone and human orthologues were studied in inside-out patches from transfected HEK-293 cells. It should be kept in mind that TRPM2 channels display divergent properties in mammalian expression systems and in frog oocytes^23^. Each patch typically contained a high number of channels, allowing to study macroscopic currents and to assess the on-kinetics (i.e. channel activation after addition of ADPR) as well as the off-kinetics (i.e. channel deactivation after removal of ADPR) in each patch (Fig. 2a–c). The respective values for τ_on_ and τ_off_ are given in Table 1. The activation of the two orthologues drTRPM2 and nvTRPM2 was fast, whereas it was significantly slower in hTRPM2. None of the orthologues showed any obvious rundown over 30 s. The typical rundown of nvTRPM2 currents in whole-cell experiments seems to be related to unknown factors not preserved in isolated patches (see also ref. ^23^). As in the case of activation, deactivation was fast in drTRPM2 and nvTRPM2 and significantly slower in hTRPM2. DrTRPM2 as well as nvTRPM2 required lower concentrations of ADPR (100 µM each) for a robust activation compared with hTRPM2 (300 µM). Further addition of ADPR did not increase activation further. Single channel conductance was assessed during the initial seconds after ADPR-application when only a few channels were active (Fig. 2d). The values were distinct for each orthologue (Fig. 2e) but again, drTRPM2 was much closer related to nvTRPM2 than to hTRPM2. The mean open time (MOT) of hTRPM2 channels is known to be extremely long, with openings frequently lasting for several seconds. This normally precludes the conventional determination of the MOT. DrTRPM2 and nvTRPM2 show long openings as well, although less extremely than hTRPM2 (Fig. 2d). Accordingly, the power spectra of macroscopic currents through drTRPM2 and nvTRPM2 were indistinguishable, whereas an enhancement of low frequencies (1 to 3 Hz) was characteristic for hTRPM2.

**Table 1:** On-off-kinetics of inside-out patch-clamp recordings in TRPM2 species variants.

	On-kinetic (τ)	Off-kinetic (τ)
hTRPM2	7.98 ± 1.90 s	8.45 ± 2.02 s
drTRPM2	0.40 ± 0.21 s***	1.42 ± 0.24 s**
nvTRPM2	0.13 ± 0.06 s***	1.46 ± 1.13 s**

Each value represents the mean ± s.d. Statistical significance was assessed using one-way-ANOVA with Bonferroni correction; n = 3. On- and off-kinetics of drTRPM2 and nvTRPM2 were significantly different compared with hTRPM2 but not among each other.

### Agonist specificity of drTRPM2 is consistent with a N-terminal ADPR-binding pocket

**Figure 3:**
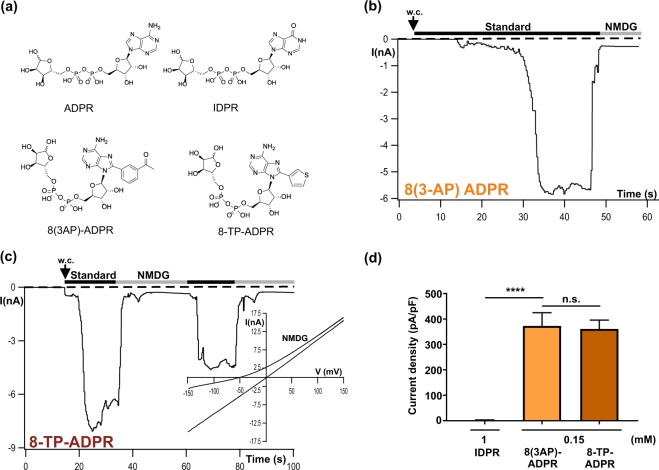
Sensitivity of drTRPM2 to the ADPR analogues 8-TP-ADPR, 8-(3AP)-ADPR and IDPR. (**a**) Structures of ADPR and several synthetic ADPR-analogues with modifications in the adenosine motif. (**b**,**c**) Representative whole-cell patch-clamp experiments of HEK-cells expressing wild-type drTRPM2. Stimulations were performed by infusion of the cells with a pipette solution containing 1 µM Ca^2+^ and either 8-(thiophen-3yl)-ADPR (0.15 mM), 8 (3-acetylphenyl)-ADPR (0.15 mM) or IDPR (1 mM) as indicated. Corresponding current-voltage relation is given as inset in panel c. (**d**) Summary of the experiments shown in panels b and c. All data are presented as mean ± s.e.m. Differences are significant at ****(P < 0.0001), evaluated with one-way ANOVA and the Bonferroni correction, n = 4–8. n.s., not significant.

For nvTRPM2 it has been demonstrated that the two ADPR derivatives 8-(thiophen-3-yl)-ADPR and 8-(3-acetyl-phenyl)-ADPR, abbreviated as 8-TP-ADPR and 8-(3AP)-ADPR, respectively, (depicted in Fig. 3a) represent effective agonists, acting on a NUDT9H-independent ADPR interaction site of the channel^9^. In contrast, the two compounds have been described as inhibitors on hTRPM2^24^ where gating essentially depends on the intact NUDT9H domain (e.g. ref. ^14^). Since such a NUDT9H-independent ADPR interaction site has been localized in drTRPM2^8^, we tested the effects of each of these ADPR analogues on wild-type drTRPM2. In whole-cell recordings of HEK-293 cells transfected with wild-type drTRPM2 we observed that both 8-TP-ADPR and 8-(3AP)-ADPR generate sizeable currents when applied to the cell (0.15 mM) through the pipette solution. These currents were almost indistinguishable from those induced by ADPR in terms of amplitudes, kinetics and I/V relation (Fig. 3b–d). Inosine 5′-diphosphate ribose (IDPR) is very similar to ADPR with only a small modification of the adenine ring (Fig. 3a). It serves as a substrate of the human NUDT9 ADPRase but only at high concentrations^25^. Recently, we demonstrated that hTRPM2 can also be activated by high concentrations of IDPR, revealing that it should be understood as a partial agonist on the NUDT9H-specific ADPR binding site. However, this does not apply to the NUDT9H-independent ADPR interaction site of nvTRPM2 where it fails to substitute ADPR as an agonist^9^. In the present study high concentrations (1 mM) of IDPR did not induce currents in HEK-293 cells expressing wild-type drTRPM2 (Fig. 3d). On a functional and pharmacological basis, these data support the conclusion of a recent cryo-EM-study^8^ that interaction of ADPR with an N-terminal binding site is the decisive step during gating of drTRPM2.

### drTRPM2 is fully functional with the NUDT9H domain of hTRPM2

**Figure 4:**
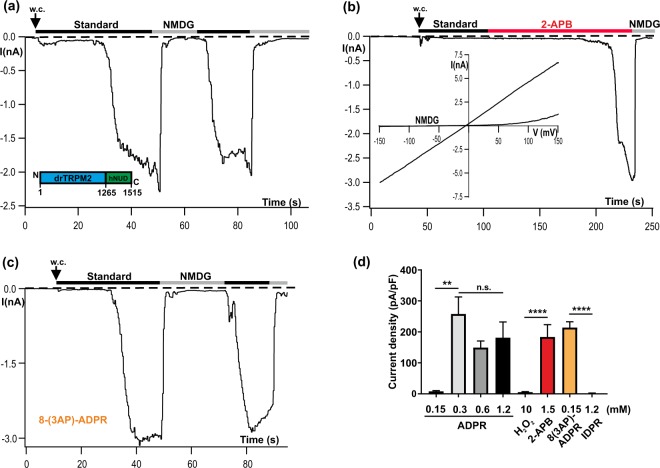
The functional properties of the chimera drTRPM2-hNUD are similar to wild-type drTRPM2. (**a**) Representative whole-cell patch-clamp experiment of a HEK-293 cell expressing drTRPM2-hNUD. ADPR (1.2 mM) was infused into the cell through the patch pipette together with 1 µM Ca^2+^. Current onset occurs with a short delay after reaching whole-cell configuration (w.c.). Substitution of external Na^+^ in the standard bath solution (black bars) with the impermeable cation NMDG (gray bars) blocks the inward currents. (**inset**) Sketch of the chimera drTRPM2-hNUD containing the NUDT9H-domain of hTRPM2. For the exact procedure see Methods. (**b**) Same experiment as depicted in panel a but stimulation was performed by superfusion of the cells with standard bath solution containing 2-APB (1.5 mM; indicated with red bar). The pipette solution contained 1 µM Ca^2+^ without ADPR. (**c**) Same experiment as depicted in panel a but stimulation was performed with 0,15 mM 8-(3AP)-ADPR together with 1 µM Ca^2+^ in the pipette solution (**d**) Summary of the experiments shown in panel a–c as well as of experiments using H_2_O_2_ as the stimulus. All data are presented as mean ± s.e.m. Differences are significant at **(P < 0.01) and ****(P < 0.0001), evaluated with one-way ANOVA and the Bonferroni correction, n = 3–8. n.s., not significant.

In spite of the prominent role of the N-terminal ADPR interaction site in drTRPM2, the NUDT9H domain is indispensable as well, because its deletion abolished channel function as previously demonstrated for hTRPM2^8,15^. In contrast, in nvTRPM2 the ADPR-induced activation occurs independently of the NUDT9H domain^15^. According to recent cryo-EM data^6,8^ the respective functional roles of the NUDT9H domain in these two vertebrate orthologues seem to be different, i.e. mere structural stabilization in drTRPM2 vs binding of ADPR with subsequent conformational changes in hTRPM2. To gain more insight into the functional role of NUDT9H in drTRPM2, we created the chimera drTRPM2-hNUD where the original NUDT9H domain of drTRPM2 was replaced by the NUDT9H domain of hTRPM2. This substitution was performed in the same way as described for hTRPM2 and nvTRPM2 in a previous study^19^. The chimera drTRPM2-hNUD (illustrated in Fig. 4a inset) displayed ADPR-induced currents (Fig. 4a) with kinetics indistinguishable to that of wild-type drTRPM2 (compare with Fig. 1a). Likewise, drTRPM2-hNUD responded in a similar manner as wild-type drTRPM2 when the stimulation was performed with 2-APB (1.5 mM; Fig. 4b). Actually the chimera drTRPM2-hNUD contains two potential ADPR-interaction sites to control ADPR-dependent channel activation. These two sites were shown to have a different pharmacological profile (N-terminally sensitive to 8-PT-ADPR and 8-(3AP)-ADPR and C-terminally sensitive to IDPR)^9^. Therefore, we tested which of these two ADPR-interaction sites controls gating in drTRPM2-hNUD. Stimulation with 8-(3AP)-ADPR (0.15 mM) causes a typical activation of the chimera (Fig. 4c), while stimulation with high concentrations (1 mM) of IDPR has no effect (Fig. 4d). A detailed analysis (Fig. 4d) revealed that there was generally a moderate reduction of the responses, which became severe in the presence of lower ADPR concentrations (compare with Fig. 1d). In line with this latter finding, there were no responses to H_2_O_2_, presumably because H_2_O_2_ acts via intracellular accumulation of ADPR^14^.

Thus, in drTRPM2 the N-terminal ADPR interaction site controls gating of drTRPM2 but the essential stabilizing function of NUDT9H can be also accomplished by the human version.

### The NUDT9H domain of drTRPM2 is sufficient for ADPR-induced gating of hTRPM2

**Figure 5:**
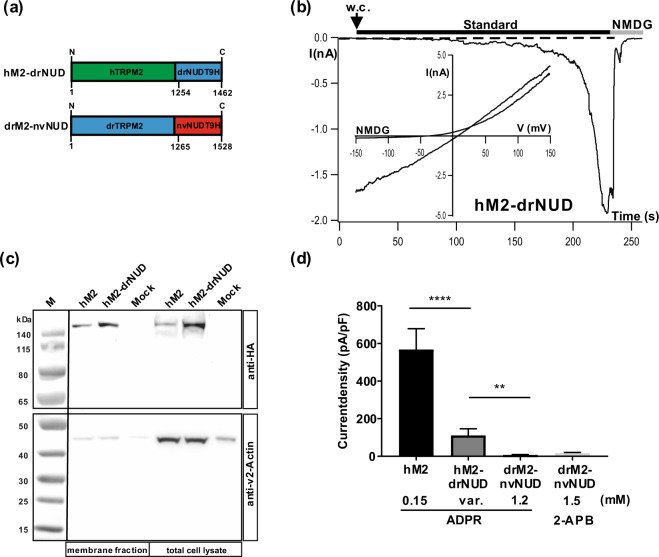
hTRPM2 is basically functional with the NUDT9H domain of drTRPM2. (**a**) Sketch of the chimeras hTRPM2-drNUD and drTRPM2-nvNUD where the NUDT9H-domains between the TRPM2 orthologues of human, zebrafish and sea anemone are exchanged as indicated. For the exact procedure see Methods. (**b**) Example for a successful stimulation of a HEK-293 cell expressing hTRPM2-drNUD with ADPR during whole-cell patch-clamp analysis. ADPR (1.2 mM) was infused into the cell through the patch pipette together with 1 µM Ca^2+^. Current develops gradually after reaching whole-cell configuration (w.c.). (**c**) Cell surface expression of wild-type hTRPM2 and of the chimera hTRPM2-drNUD was analyzed by biotinylation and subsequent reducing 4–12% SDS-PAGE. Mock-transfected cells were used as negative control. One gel was loaded with the eluted membrane proteins, another gel was loaded with the corresponding total cell lysates. PVDF membranes were cut between the marker bands for 50 and 65 kDa. The upper part was incubated with anti-HA, the lower part with anti-β-actin antibody. Different exposure times were needed due to differences in signal intensity between HA and β-actin. Wild-type hTRPM2 as well as the chimera hTRPM2-drNUD were detected in the Avidin-bound fraction representing the pool of biotinylated surface expressed proteins. Reduced β-actin staining in the membrane fraction indicates biotinylation of cytosolic proteins in damaged cells. Three independent experiments gave similar results. (**d**) Summary of the experiments investigating the channel activity of hTRPM2-drNUD and drTRPM2-nvNUD. Wild-type hTRPM2 was used as the positive control. For hTRPM2-drNUD only responders were considered which were obtained at various (var.) concentrations of ADPR. All data are presented as mean ± s.e.m. Differences are significant at **(P < 0.01) ****(P < 0.0001), evaluated with one-way ANOVA and the Bonferroni correction, n = 3–9. n.s., not significant.

Previous data on drTRPM2 suggest that its NUDT9H domain binds ADPR only weakly^6^, even though the domain is indispensable for channel function. When we constructed a chimera where hTRPM2 contains the NUDT9H-domain of drTRPM2 i.e. the chimera hTRPM2-drNUD (Fig. 5a), the absence of ADPR-induced currents was expected. However, this was clearly not the case (Fig. 5b). In this particular experiment, a typical response to ADPR was observed with regards to wild-type hTRPM2. Considering the whole experimental series, currents through hTRPM2-drNUD were found only in a minority of cells, but in this fraction, the currents were robust and reached a current density of about 30% of control (wild-type hTRPM2). In more detail, typical strong currents were observed in 15 out of 46 cells, in the presence of widely varying ADPR concentrations (0.15 to 3 mM). In contrast, currents were completely absent in the remaining cells. There was no obvious trend to more consistent responses when higher ADPR concentrations were used. As control, we followed the currents in HEK-293 cells transfected with hTRPM2-drNUD without ADPR in the patch-pipette (n = 3) and after extracellular stimulation with 10 mM H_2_O_2_ (n = 3) and found no current development. Moreover, we checked for the surface expression of hTRPM2-drNUD and confirmed that lacking expression cannot account for lack of currents (Fig. 5c).

Since in general, hTRPM2 displays an “all-or-nothing” response to ADPR almost independently of the agonist concentration, the results on the chimera are consistent with the view that the activation thresholds are higher than in wild-type but that normal channel function is preserved after the exchange of the human with the zebrafish NUDT9H domain. In strict contrast, completely dysfunctional chimeras were produced when zebrafish TRPM2 was linked to the NUDT9H domain of nvTRPM2. This chimera (drTRPM2-nvNUD) could not be stimulated with ADPR and not with 2-APB either (Fig. 5d). This result corresponds to previous data where the NUDT9H domain of nvTRPM2 was shown to be functionally incompatible with hTRPM2^19^.

### A point mutation in NUDT9H abolishes channel gating of drTRPM2 and hTRPM2

**Figure 6:**
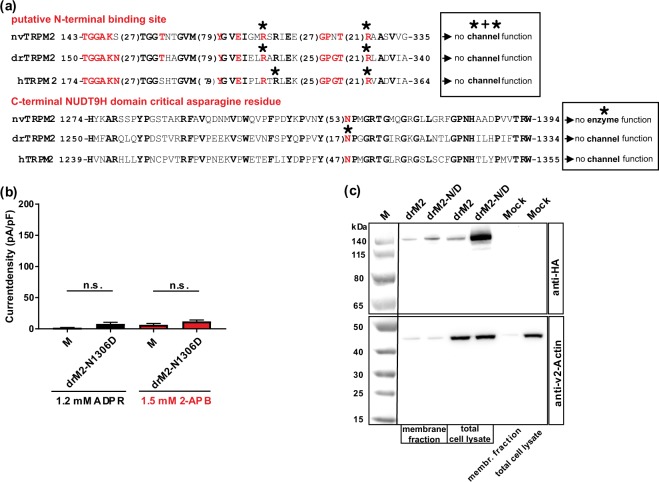
Functional importance of a highly conserved asparagine residue in NUDT9H. (**a**) Partial sequences of the TRPM2 orthologues of human, sea anemone and zebrafish encompassing the putative N-terminal ADPR-binding pocket or the N-terminal part of the NUDT9H domain (as indicated). The numbers given at the beginning and at the end of each sequence indicate the exact position within the corresponding open reading frame. Numbers in brackets specify the lengths of spacer sequences. Conserved amino acid residues are given in bold. The amino acid residues forming the novel ADPR-binding pocket as well as the critical and highly conserved asparagine residue of NUDT9H are highlighted in red. Residues which have been mutated in this study are marked with an asterisk. The two text boxes summarize functional effects of these mutations. (**b**) Summary of the experiments investigating the channel activity of a mutant of drTRPM2 where the corresponding asparagine of the NUDT9H domain was changed to aspartate (mutation N1306D). Stimulations were performed with ADPR or with 2-APB as indicated. All data are presented as mean ± s.e.m. Statistical analysis was performed with an unpaired Student’s t-test, n = 4–8. n.s., not significant. For comparison note the scale of the ordinates of Figs. 1d, 4d. (**c**) Comparison of cell surface expression of drTRPM2-N1306D (abbreviated as drM2-N/D) and wild-type drTRPM2 as indicated. Analysis was performed as described in Fig. 5c. Wild-type drTRPM2 as well as the mutant drTRPM2-N1306D were detected in the Avidin-bound fraction representing the pool of biotinylated surface expressed proteins. Reduced β-actin staining in the membrane fraction indicates biotinylation of cytosolic proteins in damaged cells. Three independent experiments gave similar results.

Besides the process of binding of ADPR, both in hTRPM2 and drTRPM2 the interaction between the NUDT9H domain and the remainder of the channel seems to play a crucial role for channel function^6,8^. Most of the previously investigated mutations of the NUDT9H domain which affected channel activity of hTRPM2 were closely associated with the catalytic function or with the binding of ADPR e.g. refs. ^14,18,26^. A remarkable exception is a highly conserved asparagine residue in the N-terminal part of NUDT9H (Fig. 6a) whose deletion or mutation to aspartate results in a loss-of-function phenotype of hTRPM2^13^. This residue is unlikely to have a direct effect on ADPR-binding^18^ and therefore should exert a function beyond binding^15^. When we tested the corresponding mutation N1306D of drTRPM2, we observed a loss-of-function phenotype since neither ADPR nor 2-APB induced channel gating (Fig. 6b). A cell surface biotinylation assay and Western-blot analysis of HA-tagged drTRPM2-N1306D confirmed that this channel mutant is adequately inserted into the plasma membrane (Fig. 6c). Thus, a strongly conserved asparagine residue in NUDT9H is crucial for the function of the two TRPM2 species variants hTRPM2 and drTRPM2, although the corresponding domains have significant structural differences and distinctly different abilities to bind ADPR^6^.

### Mutation of the novel ADPR-binding site inhibits TRPM2 function of all three species

A combination of cryo-EM-structure analyses and functional studies revealed an ADPR-binding pocket within the N-terminal part of drTRPM2^8^ (illustrated in Fig. 6a). It was demonstrated that the arginine to alanine (R/A) double mutation R278A + R334A nearly abolishes ADPR-dependent channel gating of drTRPM2 (tested with 0.1 mM ADPR + 1 mM Ca^2+^ in inside-out patches)^8^, whereas in hTRPM2 the corresponding mutation (R302A + R358A) was without effect on channel activation induced by ADPR (tested with 1 mM H_2_O_2_ in Ca^2+^-imaging experiments)^6^.

**Figure 7:**
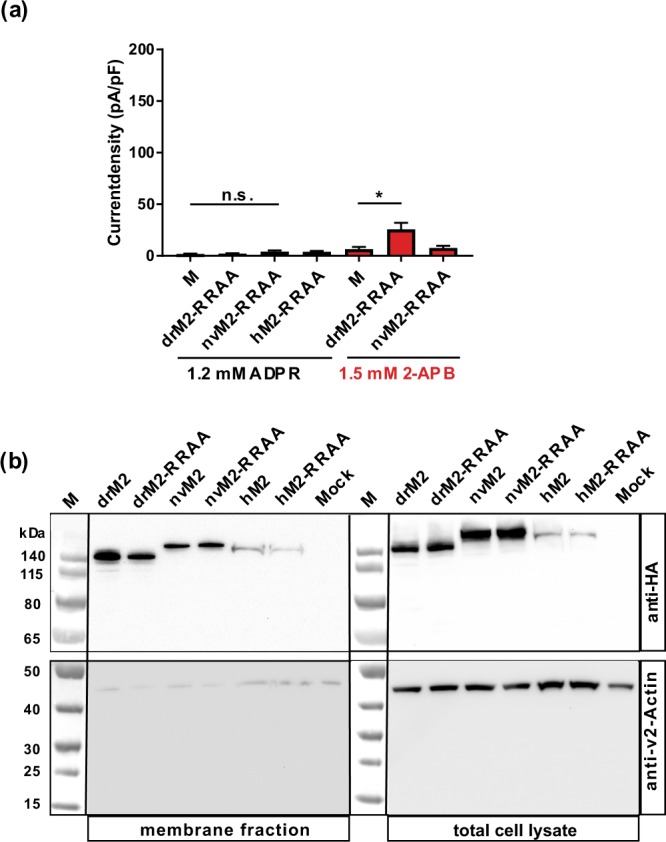
The putative N-terminal ADPR binding site is generally important for TRPM2. (**a**) Summary of the experiments investigating the channel activity of nvTRPM2, drTRPM2 and hTRPM2 after mutation of two highly conserved arginine residues of the putative N-terminal ADPR-binding pocket (see Fig. 6a) to alanines (abbreviated as RR/AA). Differences are significant at *(P < 0.05), evaluated with one-way ANOVA and the Bonferroni correction, n = 5–9. n.s., not significant. (**b**) Comparison of cell surface expression of wild-type channels and RR/AA variants for drTRPM2, nvTRPM2 and hTRPM2 as indicated. Analysis was performed as described in Fig. 5c. Since there were considerable differences in signal intensity between hTRPM2 and the two other species variants the amount of protein that was loaded onto the gel was doubled for human samples. The calculated molecular weights of full-length wild-type channels were: 175 kDa (hTRPM2-3xHA), 172 kDa (drTRPM2-3xHA) and 179 kDa (nvTRPM2–3xHA). For all investigated species variants both wild-type and mutated channels were detected in the Avidin-bound fraction representing the pool of biotinylated surface expressed proteins. Reduced β-actin staining in the membrane fraction indicates biotinylation of cytosolic proteins in damaged cells. Three independent experiments gave similar results.

We repeated this experiment with drTRPM2 under more stringent experimental conditions (1.2 mM ADPR + 1 µM Ca^2+^ in the pipette solution or extracellular 1.5 mM 2-APB; whole-cell patch-clamp analysis) and additionally tested the corresponding mutation (R271A + R329A) in nvTRPM2, because this species variant contains the N-terminal ADPR interaction site as well. The results of our experiments are summarized in Fig. 7a. For nvTRPM2-(R271A + R329A), high concentrations of either ADPR or of 2-APB failed to elicit significant currents, if compared to mock-transfected HEK-293 cells. Likewise, in drTRPM2-(R278A + R334A), there were no significant responses to high concentrations of ADPR and only miniscule currents in the presence of high concentrations of 2-APB.

Since the double R/A mutation of hTRPM2 has already been demonstrated to leave ADPR-dependent gating of hTRPM2 intact^6^, we decided to introduce a slight variation and studied the double mutant R304A + R358A instead of R302A + R358A. This alternatively mutated arginine residue at position 304 is also conserved in all three orthologues (see Fig. 6a). Unexpectedly, this double mutant hTRPM2-(R304A + R358A) also displayed a complete loss of ADPR-dependent channel activity (Fig. 7a). We did not test 2-APB because it is an inhibitor rather than an activator of wild-type hTRPM2^21,22^. Again, a cell surface biotinylation assay and Western-blot analysis of all corresponding HA-tagged channel variants revealed no important differences in the cell surface expression (Fig. 7b).

## Discussion

An N-terminal ADPR-binding pocket has been recently proposed as decisive for channel gating of the zebrafish orthologue drTRPM2^8^. Here we fully corroborate this conclusion on the basis of functional data. The pharmacological profile of drTRPM2 activation, either with ADPR, ADPR-analogues or with 2-APB, is identical with that of nvTRPM2 where ADPR binding occurs undoubtedly N-terminally and the C-terminal NUDT9H domain does not contribute to gating^15^. Moreover, our findings considerably extend the understanding of the NUDT9H domain of drTRPM2 because it is not only indispensable for gating of drTRPM2 but can also accomplish gating of hTRPM2 in the chimera hTRPM2-drNUD. On the other hand, a single point mutation within NUDT9H abolishes channel function in both, drTRPM2 and hTRPM2.

The finding that nvTRPM2 can be activated by ADPR in the complete absence of the C-terminal NUDT9H domain^15^ initiated a paradigm-shift of how ADPR gating may be achieved^27^. The well-established binding of ADPR to a catalytically inactive NUDT9H domain in hTRPM2 (e.g. refs. ^14–16^) cannot represent an universal mechanism of gating across all species. The recent structure-function analysis of drTRPM2 adds to the complexity because here, ADPR binds to a N-terminal binding pocket but gating is still impossible without the NUDT9H domain^8^.

With our chimeras of different TRPM2 orthologues, we demonstrate that the NUDT9H domains of drTRPM2 and hTRPM2 are interchangeable in generating ADPR-sensitive channels. In the chimera drTRPM2-hNUD, there is overwhelming evidence that the effective binding of ADPR occurs in the N-terminal binding pocket, because channel activation is induced by 8-(3AP)-ADPR, which exclusively acts on the corresponding site in nvTRPM2^9^.

On the other hand, functional ADPR-binding to the NUDT9H domain in drTRPM2-hNUD is unlikely because high concentrations of IDPR failed to induce channel gating (see also ref. ^9^). Most importantly, the results clearly show that the NUDT9H domain of hTRPM2 can serve as a fully functional substitute of the original NUDT9H domain of drTRPM2. Moreover this must be largely independent of its capacity to bind ADPR because the isolated NUDT9H domain of wild-type drTRPM2 binds ADPR very inefficiently^6^. The differences in the structure of the NUDT9H domain are very distinct between drTRPM2 and hTRPM2. Notably, the so-called P-loop of the NUDT9H domain supports specific (trans) inter-domain interactions as well as interactions with calmodulin in hTRPM2 but is largely absent in drTRPM2^6,20^. The more so remarkable is our identification of a highly conserved amino acid residue in the NUDT9H domain that is crucial for channel function in both vertebrate TRPM2 channels. This asparagine residue is located upstream of the P-loop region and is, in all likelihood, not directly involved in the binding or catalysis of ADPR (e.g. ref. ^18^). Therefore, this residue is probably crucial for conformational changes that follow ADPR-binding and eventually lead to gating, no matter whether ADPR-binding occurs within the NUDT9H domain as in hTRPM2 or distantly in the N-terminus as in drTRPM2.

In nvTRPM2, the role of the NUDT9H domain is different: it does not contribute to the gating process but exerts full ADPRase activity^10,15^, thereby controlling the availability of ADPR to the channel^15^. In line with this, the mutation of the critical asparagine in nvTRPM2 did not change ADPR-directed channel gating^15^. Moreover, the NUDT9H domain of nvTRPM2 could not replace the endogenous NUDT9H domains, neither in drTRPM2 as demonstrated in this study nor in hTRPM2^19^. Both vertebrate TRPM2 channels were non-functional when the original NUDT9H domains were substituted with that from nvTRPM2.

According to our results obtained with the ADPR analogs 8-TP-ADPR, 8-(3AP)-ADPR and IDPR the N-terminal ADPR-binding pockets of drTRPM2 and nvTRPM2 have the same pharmacological profile. Moreover, the mutation of homologous amino acid residues within this binding pocket suppressed ADPR-dependent channel activation of both drTRPM2 and nvTRPM2. Therefore, it can be assumed that the N-terminal ADPR-binding pockets of these two channel orthologues are largely identical. However, our data show that the mutation of a further homologous amino acid residue within this region suppresses ADPR-directed channel gating of hTRPM2 as well, although there the binding of ADPR to NUDT9H represents the crucial step in channel gating (e.g. ref. ^14–16^). Possibly, the N-terminal domain not only binds ADPR but has additional significance for channel gating. The result of our study that both in drTRPM2 and in nvTRPM2 the same double mutation that abolishes sensitivity to ADPR also suppresses activation by 2-APB, might be an indication in this direction, because 2-APB most probably acts via the pore^21^.

As important control, we checked for surface expression of all critical variants and thereby confirmed that diminished or abolished currents truly represent an impaired channel function.

On the basis of these results, the most convincing explanation would be that ADPR-dependent gating of drTRPM2 represents a cooperation of the N-terminal ADPR-binding pocket with the NUDT9H domain. Moreover and in analogy, it may be hypothesized that a similar cooperation may take place in hTRPM2 where the C-terminal NUDT9H domain with bound ADPR requires an N-terminal counterpart which is homologous to the ADPR-binding pocket of drTRPM2 and nvTRPM2 but does not necessarily bind ADPR. At this point it should be noted that there are conflicting experimental results for hTRPM2 with respect to N-terminal ADPR-binding (see ref. ^6^ vs.^28^, and the data of this study). Further experiments including new approaches are necessary to clarify the issue.

The zebrafish orthologue drTRPM2 is considerably more similar to nvTRPM2 than to hTRPM2 in many aspects, such as the pharmacological profile and the single channel properties. There is one very striking exception, and this is the kinetics of the whole cell currents that are sustained as in hTRPM2 but show rapid inactivation in nvTRPM2. These findings are in line with an interpretation that the functional roles of TRPM2 are alike in both vertebrates, in spite of the dramatic differences in ADPR binding.

In conclusion, our data indicate that TRPM2 orthologues from man, zebrafish, and sea anemone essentially require a highly conserved N-terminal domain for channel function. In dependence on the particular species, this N-terminal domain (i) binds ADPR and directly activates the channel in the sea anemone, (ii) binds ADPR and activates the channel in cooperation with the NUDT9H domain in zebrafish or (iii) possibly cooperates with the ADPR-coupled NUDT9H domain in gating of the human channel. It may be envisaged that future studies will establish universal principles of TRPM2 channel gating in response to ADPR, even if the binding of the agonist occurs in widely varying domains.

## Methods

### Molecular cloning

The cDNA of zebrafish (*Danio rerio*) TRPM2 (drTRPM2; UNP reference UPI0004F4953D)^8^ was obtained from MWG-Biotech (Ebersberg, Germany). The open reading frame (ORF) was synthesized in two parts. The 5′-terminal part consisting of 2181 bp of the ORF starts with a restriction site for *Eco RI* followed by the start codon and ends with a restriction site for *Sal I*. The 3′-terminal part consisting of 2262 bp of the ORF starts with a restriction site for *Sal I* and ends with a stop codon followed by a restriction site for *Xba* I. The codon usage of the synthesized cDNA was modified for optimal expression in human cells (HEK-293 cells). The correct sequence of the synthesized cDNA was checked with double-strand DNA sequencing performed by MWG-Biotech. The 5′-terminal part of the ORF was subcloned via *Asc* I + *Sal I* into the modified pIRES-hrGFP-2a vector (Stratagene, La Jolla, CA, USA) that contains a unique *Asc* I site instead of a single *Nhe* I site as well as a unique *Xba* I site instead of a single *Xho* I site. Subsequently, the 3′-terminal ORF was added via a *Sal I* + *Xba* I cloning step. The cDNAs of hTRPM2 and nvTRPM2 were subcloned as described previously^19^. Site-directed mutagenesis was performed using the QuikChange mutagenesis system (Agilent, Santa Clara, CA, USA). Defined oligonucleotides in sense and antisense configuration were obtained from MWG-Biotech. Chimeras of drTRPM2, hTRPM2 and nvTRPM2 were generated by exchanging the corresponding NUDT9 homology (NUDT9H) domains. The swapped NUDT9H sequences were as follows: (drTRPM2: aa 1266–1474¸hTRPM2: aa 1253–1503; nvTRPM2: aa 1289–1551). Exclusively for the specific immunohistological detection in surface expression experiments and Western-blot analysis, wild-type, mutant and chimeric channels were C-terminally fused with a triple hemagglutinin (3xHA)-tag. This was performed as described previously^15^. Each chimeric channel construct as well as all point mutations were checked by DNA sequencing (MWG-Biotech). Unless indicated otherwise, all procedures were performed in accordance to the respective manufacturer’s instructions.

### ADPR-analogues

Synthetic ADPR analogues inosine 5′-diphosphate ribose (IDPR), 8-(thiophen-3-yl)-adenosine 5′-diphosphate ribose (8-TP-ADPR), and 8-(3-acetyl-phenyl)-adenosine 5′-diphosphate ribose (8-(3AP)-ADPR) were prepared as previously described^9,24^.

### Cell culture and transfection

Human embryonic kidney (HEK-293) cells were purchased from the German Collection of Microorganisms and Cell Cultures (Braunschweig, Germany). Cell culture was carried out in DMEM media (Biochrome, Berlin, Germany) supplemented with 4 mM L-glutamine, 2 mM sodium pyruvate and 10% (v/v) foetal calf serum (Biochrome). Wild-type or mutant TRPM2 channels were heterologously expressed in HEK-293 cells after transient transfection of the corresponding cDNA using the FuGene 6 transfection reagent (Roche, Mannheim, Germany) according to the manufacturer’s protocol. The transfected cells were incubated for 24 h at 37 °C and 5% CO_2_. Afterwards, the cells were harvested for biotinylation assay and Western blot analysis. Alternatively, the cells were seeded on poly-lysine-coated glass coverslips at a suitable dilution and further incubated for 3–4 h. Then, patch-clamp experiments were performed with cells visibly positive for EGFP-expression. At least three independent transfections were used for each experimental group.

### Cell surface biotinylation and Western-blot analysis

Biotinylation assays were performed using Pierce Cell Surface Protein Isolation Kit (Thermo Fisher Scientific, USA) according to the manufacturer’s instruction. Therefore, two 100 × 20 mm culture dishes of transfected, sub-confluent (80%) HEK-293 cells were biotinylated and lysed in Cell Lytic M (Sigma Aldrich, USA) lysis buffer. All channel variants transfected for cell surface biotinylation and Western-blot analysis contained a C-terminally attached 3xHA-tag. Mock-transfected cells were used as negative control. Biotin concentration was adapted to cell confluence (0.2 mg/ml) and protein concentration was determined using Bradfort reagent (Sigma Aldrich, USA) according to manufacturer’s instructions. 600 µg lysed sample were incubated with NeutrAvidin beads and afterwards eluted with SDS sample buffer (Thermo Scientific, USA).

Biotinylated samples and corresponding total cell lysates were subjected to reducing SDS-PAGE (Bolt 4–12% Bis-Tris Plus Gel, Invitrogen) and Western-blot analysis. Volumes that were loaded onto the gel were doubled for human variants of TRPM2 due to lower expression levels. PVDF Membranes were cut in halves between the marker bands for 50 and 65 kDa. The upper part of the membrane was incubated with a primary monoclonal mouse-anti-HA antibody (1:1000; Sigma-Aldrich, USA) to determine TRPM2 expression and the lower part of the membrane was incubated with mouse-anti-β-actin antibody (1:1000; Cell Signaling, USA) to determine β-actin expression. Both membrane parts were incubated with a rabbit-anti-mouse-HRP conjugated secondary antibody (1:1000; DAKO, Agilent, USA). Detection of antibodies was performed using Intas Infinity ECL Starlight (Intas, Germany) and ChemoStar Touch (Intas, Germany) imaging system. For comparison, multiple exposures of the Western-blots shown in this study are given in Supplementary Figure S1.

### Electrophysiology

Whole-cell patch-clamp analysis of transfected and non-transfected (control) HEK-293 cells was carried out using an EPC 9 amplifier controlled by a personal computer equipped with Pulse 8.5 and X Chart software (HEKA, Lamprecht, Germany). The extracellular solution (referred to as standard bath solution) contained (in mM) 140 NaCl, 1.2 MgCl_2_, 1.2 CaCl_2_, 5 KCl, 10 HEPES, pH 7.4 (titration performed with NaOH). Sodium-free extracellular solution was prepared by substituion of 140 mM NaCl with 150 mM N-methyl-D-glucamine (NMDG) in the standard bath solution (titration performed with HCl). The intracellular (pipette) solution contained (in mM) 145 CsCl, 8 NaCl, 2 MgCl_2_, 10 HEPES, pH 7.2 (titration performed with CsOH) and the Ca^2+^ concentration was adjusted to to 1 µM (0.886 mM Ca^2+^, 1 mM Cs-EGTA). The Ca^2+^ concentration of the pipette solution was calculated with the *MAXC*-program: (http://www.stanford.edu/~cpatton/maxc.html). For the stimulation of TRPM2, Adenosine diphosphate ribose (ADPR; 100 mM stock solution in distilled water) was added to the pipette solution yielding final concentrations of between 0.15 and 1.2 mM. Alternatively, TRPM2 currents were evoked by superfusion of the cells with standard bath solution containing 10 mM H_2_O_2_ (diluted from a 30% stock solution). 2-Aminoethoxy diphenyl borate (2-APB; Sigma-Aldrich) was prepared as 100 mM stock solution in dimethyl sufloxide (DMSO), and aliquots kept at −20 °C. 2-APB was applied to the extracellular solution in the appropriate concentration on the day of the experiment. All experiments were performed at room temperature (21 °C) and the current-voltage relations were obtained during voltage ramps from −150 to +150 mV and back to −150 mV applied over 200 ms. The holding potential was −60 mV. For functional analysis and quantification the maximum current amplitudes (pA) obtained from a single cell were divided by the corresponding cell capacitance (pF), a measure of the cell surface. The result is the current density (pA/pF).

Inside-Out Patch Clamp recordings were performed using an Axopatch 200B amplifier and digitised via a Digidata 1440 A. Data was recorded on a personal computer with Clampex 10.7 and analysed using Clampfit 10.7 (Axon Instruments, Foster City, USA). The standard bath solution contained (in mM) 145 CsCl, 8 NaCl, 2 MgCl_2_, 10 HEPES, pH 7.2 (CsOH) and the Ca^2+^ concentration was adjusted to 1 µM. The pipette solution contained (in mM) 140 NaCl, 1.2 MgCl_2_, 1.2 CaCl_2_, 5 KCl, 10 HEPES, pH 7.4. For the application of ADPR a rapid solution exchange was performed via an RSC-200 (Bio-Logic Science Instruments, Seyssinet-Pariset, France). Patch pipettes were made of borosilicate glass (Hilgenberg, Malsfeld, Germany) and had tip resistances between 2 and 4 MΩ. A gap-free acquisition mode was used with analogous filtering at 2 kHz. Single channel conductances were estimated from fits of Gaussian functions to all-points histograms. Current kinetics (τ) were fitted by single exponential functions using nonlinear least-squares methods. The power spectrum was performed via the Clampfit 10.7 software.

### Data analysis and statistics

Unless statet otherwise, the comparison of two groups was performed using an unpaired Student’s t-test and the data are expressed as the mean ± s.e.m. Multiple comparisons were performed using an one-way ANOVA and the Bonferroni correction. Differences were considered significant at **P < 0.01, ***P < 0.001 and ****P < 0.0001.

## Supplementary information

Supplementary Figure S1

## Data Availability

The datasets generated during and/or analysed during the current study are available from the corresponding author on reasonable request.
