# Supplementary material for: Functional importance of NUDT9H domain and N-terminal ADPR-binding pocket in two species variants of vertebrate TRPM2 channels
Source: Sci Rep. 2019 Dec 16;9:19224. doi: 10.1038/s41598-019-55232-5 (PMC6914804; doi:10.1038/s41598-019-55232-5)

SUPPLEMENTARY INFORMATION

ARTICLE TITLE: Functional importance of NUDT9H domain and N-terminal ADPR-binding pocket in two species variants of vertebrate TRPM2 channels

AUTHORS: Frank J. P. Kühn*, Wiebke Ehrlich, Daniel Barth, Cornelia Kühn & Andreas Lückhoff

*Corresponding author: PD Dr. Frank Kühn, Institute of Physiology, RWTH Aachen. Email: fkuehn@ukaachen.de. Telephone +49 (0) 241 8088803

AUTHOR AFFILIATION: Institute of Physiology, Medical Faculty, RWTH Aachen, D52057 Aachen, Germany.

SUPPLEMENTARY INFORMATION LEGEND

Figure S1. Identical Western-blots as shown in Figs. 5c, 6c and 7b of the manuscript but developed with a different exposure time.

ref. to Fig. 5c

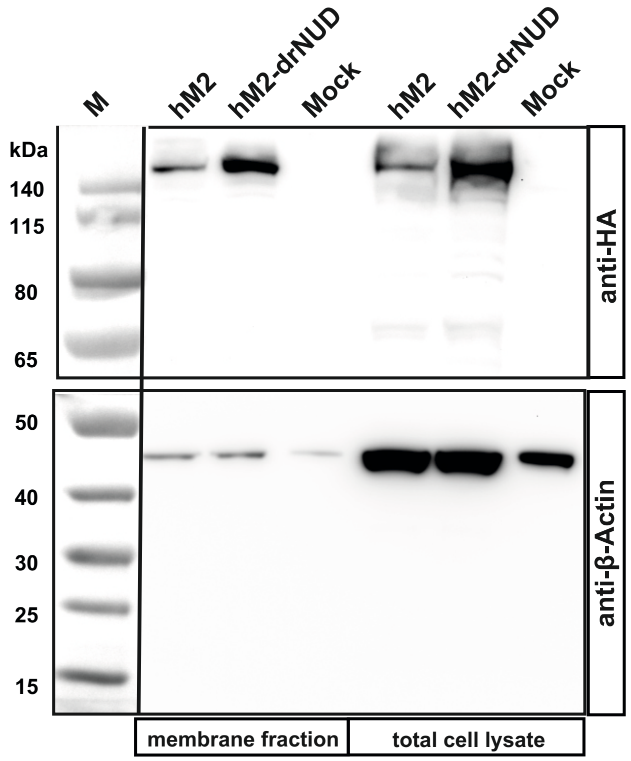

ref. to Fig. 6c

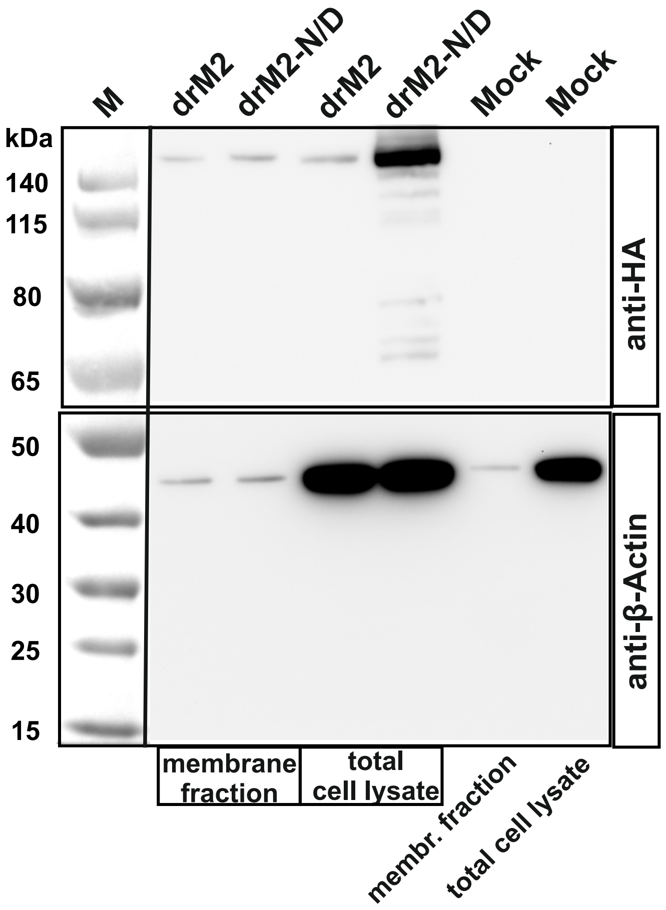

ref. to Fig. 7b

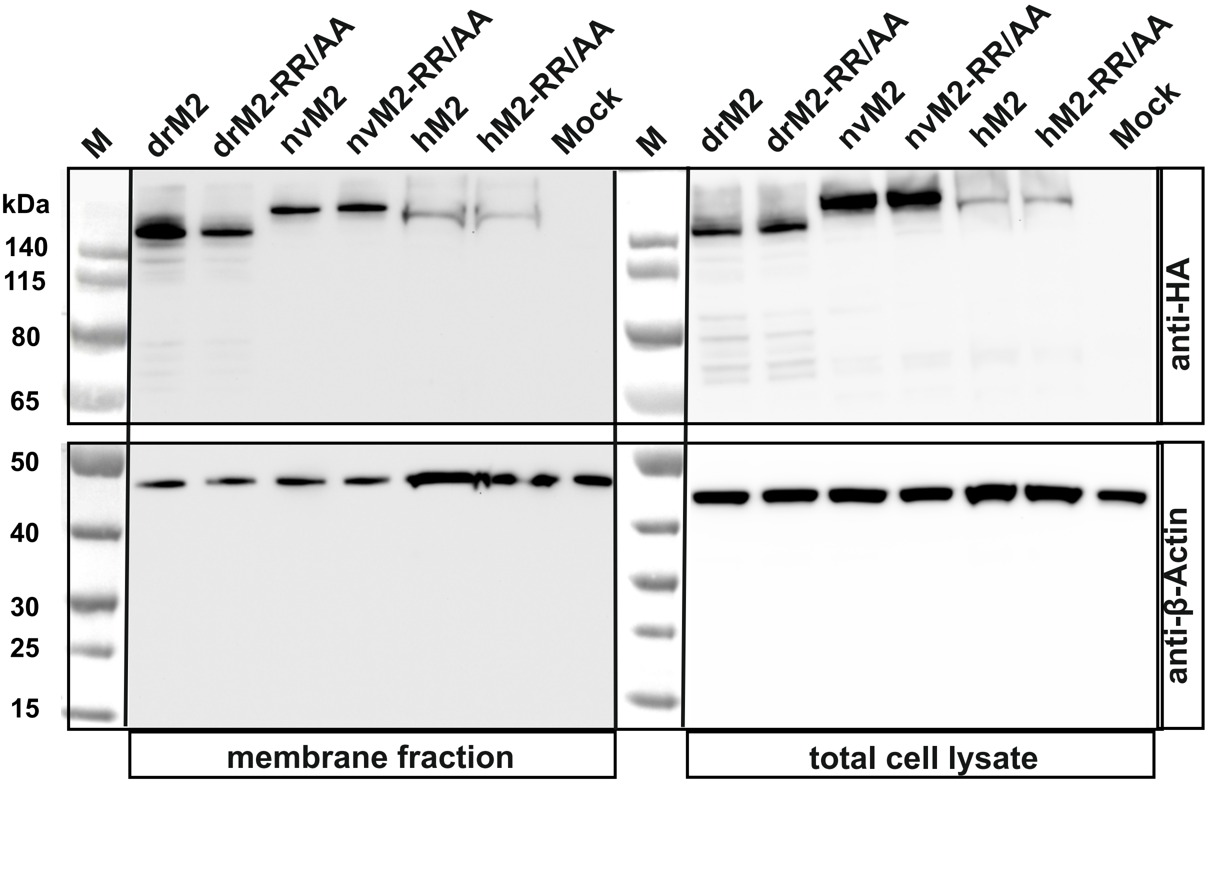

Supplement: Supplementary file 1 — Supplementary Figure S1 [file 41598_2019_55232_MOESM1_ESM.pdf]
